# Effect of Sizing Agent on the Mechanical, Thermal, and Electrical Performance of Basalt Fiber/Epoxy Composites

**DOI:** 10.3390/polym14173533

**Published:** 2022-08-28

**Authors:** Long Ma, Xiaotao Fu, Cong Zhang, Lincong Chen, Xiaolin Chen, Chuanfu Fu, Yunfei Yu, Hechen Liu

**Affiliations:** 1 Key Laboratory of Physical and Chemical Analysis for Electric Power of Hainan Province, Hairui Road No. 23, Haikou 570100, China; 2 Hebei Key Laboratory of Green and Efficient New Electrical Materials and Equipment, North China Electric Power University, Yonghua North Street No.619, Baoding 071003, China

**Keywords:** basalt fiber, sizing agent, epoxy resin emulsion, polyurethane emulsion

## Abstract

Basalt fiber and its resin composites have gradually supplanted traditional steel and glass fiber composites due to their superior strength, heat resistance, and corrosion resistance. However, basalt fiber still has significant flaws that restrict the functionality and use of its composites, such as less active functional groups and poor resin adherence. This study examines the effects of sizing agent on the characteristics of basalt fiber/epoxy resin composites. Epoxy resin emulsion and acrylate emulsion are employed as the primary auxiliary film-forming agents in this study. Polyurethane emulsion with various content levels is also used. The findings indicate that a 1% wt. of polyurethane emulsion concentration produces the greatest results, increasing the composite’s flexural strength, flexural modulus, tensile strength, and interlaminar shear strength by 122%, 34.0%, 102%, and 10.2%, respectively. At the same time, the storage modulus and Tg of the material will decrease. In addition, the breakdown strength can be raised by 112%, and insulation parameters such as leakage current and dielectric loss factor can be decreased by 26.4% and 15.6%, respectively. The effect of sizing agent B is the best.

## 1. Introduction

Basalt fiber (BF) is a brand-new class of inorganic fiber that uses natural basalt ore as its primary raw material and has high strength, high modulus, corrosion resistance, high temperature, and other features [1,2,3]. Compared to glass and carbon fiber, basalt fiber has a cheaper cost of manufacture. Basalt fiber is particularly environmentally benign and may be directly reintroduced to nature after being discarded. As a result, this fiber is receiving more and more attention from academics and professionals [4,5,6]. However, because of its flat and inert surface, basalt fiber bonds poorly with the matrix material, severely impeding the development of basalt-fiber-reinforced polymers (BFRP) [7].

Currently, surface treatment generally improves the flaw in basalt fiber. Acid-base etching modification, coupling agent modification, plasma modification, and surface coating modification are the main categories of surface modification techniques [8,9,10,11,12]. The main component of the surface coating method is the application of a layer of sizing agent for modification on the surface of basalt fiber, which can not only enhance fiber clustering and lessen fiber wear during production, but also optimize various fiber surface characteristics and improve the interaction between the fiber and the matrix. The film-forming agent, the coupling agent, the lubricant, and the antistatic agent are the major components of the sizing agent. Through orthogonal studies, Xing et al. discovered that the film-forming agent is the element that has the largest influence on the functionality of the sizing agent [13]. Additionally, there are four different types of film-forming agents: epoxy resin emulsion, polyurethane emulsion, acrylate emulsion, and polyester emulsion. In a comparison of the effects of the aforementioned four emulsions as film-forming agents on the characteristics of basalt fibers, Chu et al. of the Harbin Institute of Technology discovered that epoxy emulsion had good clustering and increased fiber breaking strength, while polyurethane emulsion could increase fiber toughness [14]. By combining SiO_2_ and modifying epoxy resin to create an organic/inorganic nano hybrid sizing agent, Wei et al. discovered that the sizing agent may increase the tensile strength and interlayer shear strength of basalt fiber bundles by 15% and 10%, respectively [15]. By adjusting the amount of the hydrophilic chain extender N-methyldiethanolamine (DMEA), Zuo et al. were able to create a consistent and stable aqueous polyurethane that they then employed as the primary film-forming component of a wetting agent to treat basalt fibers. According to the findings, the treated basalt fibers’ breaking strength could be raised to 175%, and their acid and alkali corrosion resistance was also greatly improved [16].

In order to improve the heat resistance of basalt fibers, Wang et al. synthesized a water-soluble polysiloxane sizing agent and applied it to coat basalt fibers. TG-DSC results showed that polysiloxane began to decompose at 370 °C. According to the mechanical test, coated basalt fiber can retain a breaking force of more than 76% at 300 °C, which is 3.8 times that of unsized fiber, and more than 49% at 400 °C, which is 2.4 times that of unsized fiber [17]. Jia et al. used five thermoplastic sizing agents with different chain structures to improve the interfacial properties of unidirectional BF-reinforced soluble and high-temperature-resistant poly (phthalazinone ether nitrile ketone) (BF/PPENK) composites. DMA results showed that the storage modulus and Tg of BF/PPENK composites were as high as 50 GPa and 288 °C, respectively. In addition, the tensile strength, compressive strength, bending strength, and interlaminar shear strength of BF/PPENK composite reached 778 MPa, 600 MPa, 1115 MPa, and 57 MPa, which increased 42%, 49%, 20% and 30%, respectively, compared with the unsized BF / PPENK composite [18]. Chen et al. prepared an epoxy Pickering emulsion with cationic surfactant CTAC-modified fumed silica to treat basalt fibers. It was found that the composite improved to varying degrees in terms of surface energy, interfacial shear strength, and impregnated yarn tensile strength [19].

Special sizing agents for basalt fiber research and development are still in need in China. The result is frequently unsatisfactory when manufacturers size basalt fiber directly using glass fiber sizing agents. Therefore, it is crucial to research the unique wetting agent for basalt fiber to enhance the overall properties of basalt fiber composites. Acrylate emulsion, polyurethane emulsion, and epoxy emulsion were used as auxiliary film-forming agents in this study. By adjusting the concentration of polyurethane emulsion in the auxiliary film-forming agent, three different types of specific wetting agents for basalt fiber were created. Studying the composites’ thermal, mechanical, and insulating properties, they were made by hot pressing treated basalt fiber and epoxy resin together.

## 2. Materials and Methods

### 2.1. Materials

The experimental materials used in this paper are: basalt fiber plain fabric with monofilament diameter 13 ± 1 μm and area density 300 ± 15 g/m^2^ (Sichuan Aerospace Tuoxin basalt Industry Co., Ltd, Chengdu, China); epoxy emulsion for sizing agent NBR-290, polyurethane emulsion NBR-7-1, lubricants NBR-1090 and NBR-1140, and antistatic agent NBR-1270 (Nanjing Glass Fiber Research Institute, Nanjing, China); acrylate emulsion CTD-6910 (Changzhou gene Chemical Co., Ltd, Changzhou, China); Silane coupling agent KH560 (purity ≥99%) (Jinan xingfeilong Chemical Co., Ltd, Jinan, China); Acetic acid, analytical pure (Chengdu Kelong Chemical Reagent Factory, Chengdu, China); bisphenol A epoxy resin DGEBA, epoxy value 0.51–0.54 eq/100g, epoxy equivalent 184–195 g/eq industrial pure (pelim Electric Technology Co., Ltd, Quzhou, China); methylhexahydrophthalic anhydride MHHP, 2,4,6-tris (Dimethylaminomethyl) phenol DMP-30, purity ≥95% (Guangzhou Desheng Chemical Co., Ltd, Guangzhou, China). 

### 2.2. Preparation of Samples

#### 2.2.1. Preparation of Sizing Agent

Acetic acid was added dropwise to a specified volume of deionized water to bring the pH level to between 3 and 4, then coupling agent was slowly added while the solution was vigorously stirred. Stirring continued for two to three hours after the dropwise addition until there was no evident oil. The pH of the solution was maintained at 5 to 6 so that it was ready for use after hydrolysis.

The appropriate quantity of film-forming agent emulsion was diluted approximately four times before usage. Lubricants NBR-1090 and NBR-1140 and antistatic agent NBR-1270 were dissolved in deionized water at 70 °C.

The diluted film-forming agent was poured into the container and mixed evenly with mechanical stirring. Next, additives were added such as lubricant and antistatic agent in turn, followed by the hydrolyzed coupling agent. After the mixture was well combined, it was stirred for approximately one hour at a medium speed to produce the necessary sizing agent.

Three different types of unique wetting agents for basalt fiber were produced in this study. The specific components and contents are shown in Table 1.

#### 2.2.2. Sizing Treatment of Basalt Fiber

BF was placed in a Soxhlet extractor containing acetone and the extraction process took 12 h. In this way, the chemical residues on the fiber surface could be washed off. It was put in a vacuum drying oven and dried at 50 °C for 10 h after being thoroughly cleaned with deionized water five times. The dried BF was soaked in the sizing agents A, B, and C for 1h and then dried in the drying oven at 70 °C for 5h. BFs treated with infiltrating agents A, B, and C were named A-BF, B-BF, and C-BF, respectively.

#### 2.2.3. Preparation of Basalt Fiber/Epoxy Resin Composites

DGEBA, MHHPA, and DMP-30 were blended at a mass ratio of 100:75:0.3 and then stirred in a planetary stirrer for 2 min before defoaming. The modified BF was immersed in the prepared epoxy resin solution, and the BF/epoxy resin composite was formed by hot pressing. The composites prepared by A-BF, B-BF, C-BF and epoxy resin were named A-BFRP, B-BFRP, and C- BFRP, respectively.

### 2.3. Methods

#### 2.3.1. SEM Morphologies

A length of fiber was cut, adhered to a copper sheet, and then placed on the test disk. Since basalt fiber is non-conductive, it is required to coat the sample’s surface with conductive film, or spray gold on the continuous basalt fiber, in order to prevent the migration of positive ions to negative centers brought on by charge accumulation and to enhance image quality. After vacuum pumping and gold spraying, the surface morphology of basalt fiber was characterized and analyzed. The acceleration voltage was 10kV, and the sample was amplified 1500–5000 times.

#### 2.3.2. Thermal and Mechanical Tests

Bending test: in accordance with GB/T 1449-2005, the bending test was conducted using the three-point bending method, with a 2 mm/min test speed.

Tensile test: a tensile test was performed in accordance with GB/T 1447-2005 at a test speed of 10 mm/min and using a type I cut for the sample.

Interlaminar shear test: an interlayer shear test was performed in accordance with GB/T 1450.1-2005, with a 2 mm/min test speed.

Dynamic thermomechanical analysis (DMA): the temperature was raised from room temperature to 250 °C using the single cantilever test mode at a constant rate of 5 °C/min, with a frequency of 2 Hz and an amplitude of 10 m.

#### 2.3.3. Insulating Property Test

Breakdown strength: in accordance with GB/T 16927.1-2011, a spherical electrode with diameter of 20 mm and a short-time voltage boosting method with boosting speed of 2 kV/s were used in the experiment.

Leakage current: The digital leakage current measuring device DM3068 was used to sample the composite materials. The sampling frequency was 20 Hz, the test voltage was 12 kV, and the boosting rate was 1 kV/s.

Dielectric loss factor tan*δ*: an Agilent-4294-A impedance analyzer was used according to GB/T 1409-2006, with a 5 kV test voltage.

## 3. Results and Discussion

### 3.1. SEM Analysis

**Figure 1:**
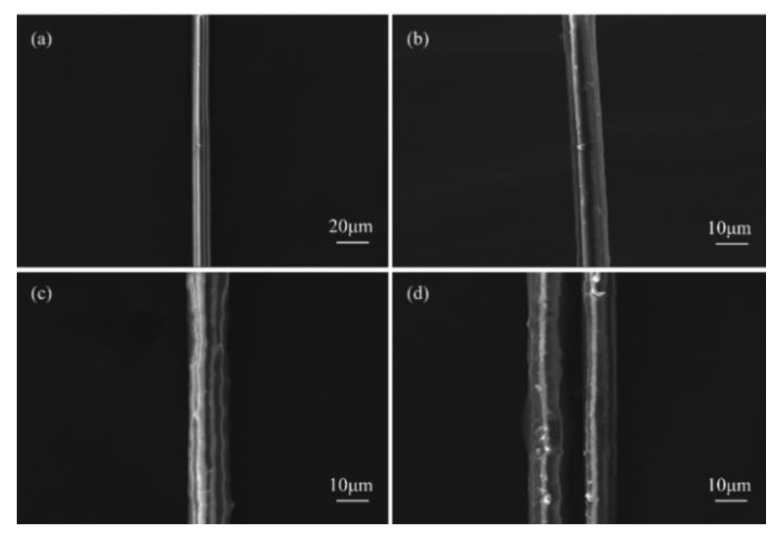
SEM of basalt fiber, (**a**) BF; (**b**) A-BF; (**c**) B-BF; (**d**) C-BF.

SEM is used to observe and research the surface morphology of basalt fiber both before and after sizing agent treatment. The results are shown in Figure 1. Figure 1a shows the untreated basalt fiber, which has a surface that is straight and smooth. Figure 1b shows the basalt fiber treated with the sizing agent A. At this time, the polyurethane emulsion ha not yet been added, and the sizing agent concentration is currently low. The surface of the basalt fiber is coated with a little amount of sizing agent. The basalt fiber sized using agent B is depicted in Figure 1c; there is a relatively obvious sizing agent film on the fiber surface, indicating that the sizing agent B has a good adhesion effect on the fiber. The basalt fiber with sizing agent C treatment is shown in Figure 1d. The thickening of the sizing agent coating on the fiber surface at this point is a result of the polyurethane emulsion’s increased concentration. The binding between the fibers is noticed at the same time, pointing to a significant rise in fiber clustering.

### 3.2. Thermal and Mechanical Properties

#### 3.2.1. Bending Properties

**Figure 2:**
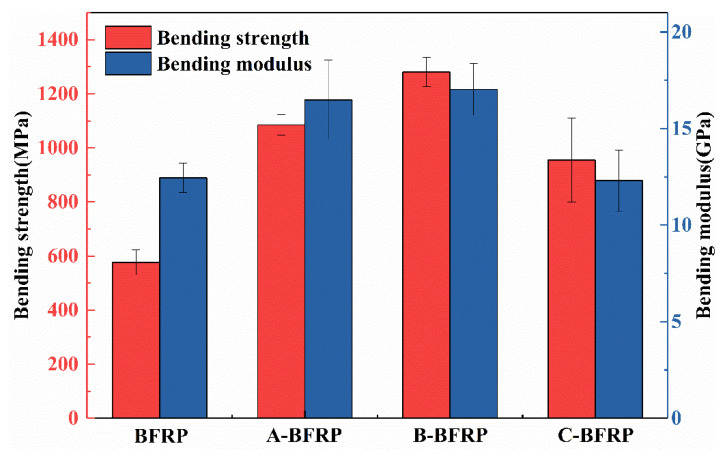
Bending properties of basalt fiber/epoxy resin composites.

The bending strength and bending modulus of the four composite materials are shown in Figure 2. When compared to untreated materials, the bending strength and bending modulus of composite materials treated with the sizing agent are both improved to varying degrees. This is primarily due to the epoxy emulsion being the main component of the film-forming agent, which has good compatibility with the epoxy resin matrix, so that the treated basalt fiber is better wetted by the resin matrix, thus improving the bending performance of the composite. The bending strength and bending modulus of B-BFRP have seen the largest increases out of all of them, rising by 703.76 MPa and 4205.15 MPa compared with the control group, with a degree of improvement of 122% and 34%, respectively. This demonstrates that with the addition of polyurethane emulsion, the sizing agent can help improve the wettability of basalt fibers. However, if the polyurethane emulsion concentration is too high, the clustering between the fibers will be too strong and will make it difficult for the resin to penetrate, which would decrease the bending performance of composites.

#### 3.2.2. Tensile Properties

**Figure 3:**
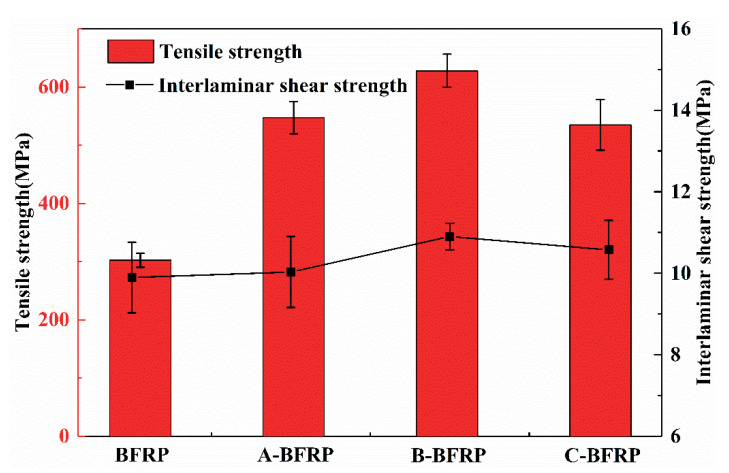
Tensile and interlaminar shear properties of basalt fiber/epoxy resin composites.

The tensile properties of basalt fiber/epoxy resin composites are shown in Figure 3. Compared with sample BFRP, the tensile strength of the composite after sizing agent treatment is significantly improved. When the concentration of polyurethane emulsion is 1 wt%, the tensile strength of B-BFRP reaches 611 MPa, which is 102% more strong than untreated BFRP. This is because the sizing agent forms a film with certain strength on the surface of the basalt fiber, and the grooves or peaks on the film are filled by the resin after compounding, resulting in a strong mechanical anchoring effect that can significantly improve the composite’s ability to withstand external loads [20].

#### 3.2.3. Interlaminar Shear Properties

The interlaminar shear of basalt fiber/epoxy resin composite is shown in Figure 3. The interlaminar shear strength of A-BFRP without polyurethane emulsion was 10.03 MPa, which is not significantly changed compared with 9.89 MPa for BFRP. In comparison to BFRP, the interlaminar shear strength of B-BFRP and C-BFRP increased by 10.2% and 6.9%, respectively. This indicates that sizing agents containing polyurethane emulsion can increase fiber toughness, make it easier for resin to immerse in it, and strengthen the bonding strength of the composite interface. Additionally, during the molding process of basalt fiber and epoxy resin, the organic end of the coupling agent in the sizing agent can react with the epoxy resin, causing the interface between the two materials to be more compact, the reinforcement effect to be more noticeable, and the interlaminar shear strength to be higher.

#### 3.2.4. DMA

**Figure 4:**
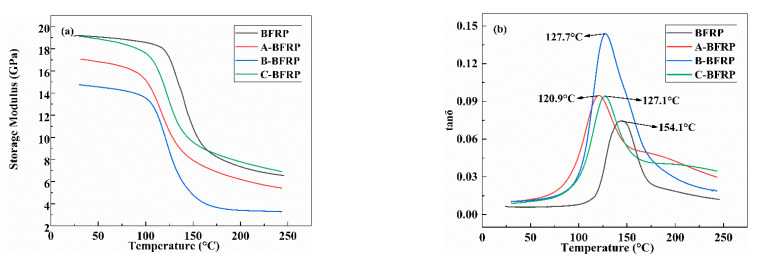
DMA of basalt fiber/epoxy resin composites: (**a**) storage modulus; (**b**) tan*δ*.

The samples were put through a dynamic thermomechanical analysis, and the results are displayed in Figure 4 along with each sample’s storage modulus and tan*δ*. As shown in Figure 4a, as the temperature rises, the storage modulus of the composite material rapidly declines. This is because when the temperature is very low, the energy is also very small, which is insufficient to overcome the main chain’s internal rotational potential barrier and leaves the molecular segments in a frozen state. Furthermore, the sizing agent containing polyurethane emulsion lowers the storage modulus of the composite to varying degrees. This is equivalent to adding a layer of polyurethane to the composite’s interface, which increases the material’s viscosity and decreases its elasticity and, in turn, reduces the storage modulus. At the same time, the decrease in the elasticity of the specimen is consistent with the increase in the initial modulus of mechanical properties [21].

The glass transition temperature of the composite is represented by the abscissa of the tan*δ* peak in Figure 4b. The glass transition temperature of the sample decreases after sizing agent treatment, which could be due to the sizing agent’s low Tg. At the same time, the increase in the peak of the loss factor indicates that there is a good interface effect between the sizing agent and the resin matrix, which makes the continuous movement of amorphous molecules in the composite more restricted, thus increasing energy consumption and the loss factor [22,23].

### 3.3. Insulating Properties

#### 3.3.1. Breakdown Strength

**Figure 5:**
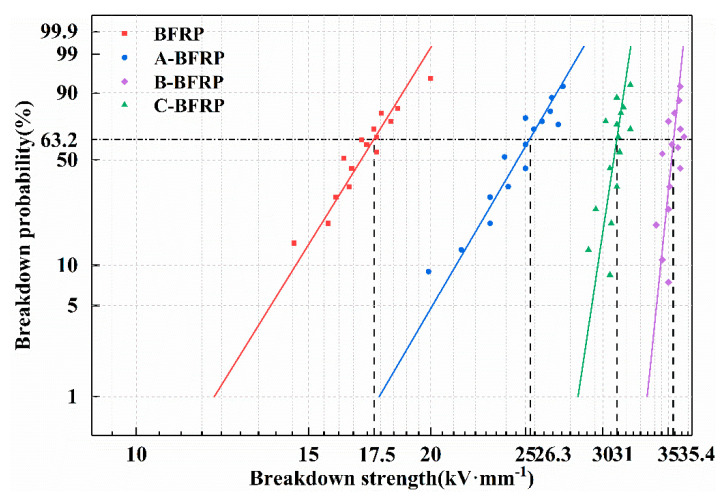
Breakdown strength of basalt fiber/epoxy resin composites.

The results of the Weibull analysis on the breakdown strength of the composite are shown in Figure 5. The breakdown voltage of the composites treated with the sizing agent is higher than that of the untreated ones. Among them, B-BFRP has the highest breakdown strength, reaching 34.64 MPa, 112% higher than BFRP’s 16.34 MPa, while C-BFRP has a slightly lower breakdown strength. This is because the sizing agent forms a layer of film on the surface of basalt fiber, which improves the interfacial bonding strength of basalt fiber and epoxy resin. Moreover, polyurethane emulsion can further improve the sizing property of the fiber and the resin matrix, repair the interface defects of the composite, and lead to the improvement of its overall breakdown strength. The breakdown voltage of the sample will, however, decrease to some level if the content of polyurethane emulsion is too high because it has a poorer insulating property than basalt fiber.

#### 3.3.2. Leakage Current

**Figure 6:**
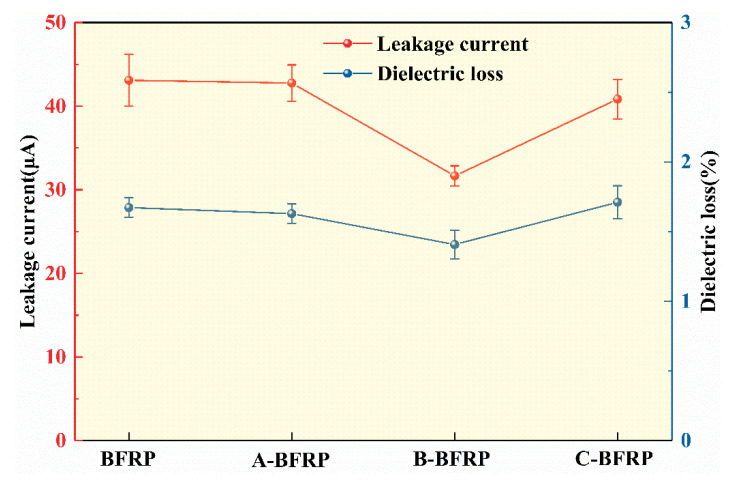
Leakage current and dielectric loss of basalt fiber/epoxy resin composites.

Leakage current experiments were carried out on four samples, and the results are depicted in Figure 6. All samples have less than 50 μA of leakage current when tested at a test voltage of 12 kV. The leakage currents of A-BFRP and BFRP are not significantly different, while those of B-BFRP and C-BFRP are relatively small. The leakage current of B-BFRP is 26.4% lower than that of BFRP, which is 31.658 μA, and the leakage current of the composite material has a strong relationship with the interface bonding degree. When the interface bonding degree is poor, the charge is easy to form a transport channel in the material, resulting in a large leakage current; however, when the interface bonding is good, the formation of charge transport channels will be suppressed, thus the leakage current can be suppressed [24]. According to the experimental findings, sizing agent B has the best effect on increasing the bonding strength of the basalt fiber/epoxy resin interface.

#### 3.3.3. Dielectric Loss

Dielectric loss tests were carried out on all basalt fiber/epoxy resin composite samples, and the results are shown in Figure 6. Since there is little variation in the dielectric loss factor across all samples, the inaccuracy is also small. The dielectric loss factors of samples BFRP, A-BFRP, and B- BFRP are not much different, which are 1.67%, 1.63%, and 1.71%, respectively; B-BFRP has a dielectric loss factor of 1.41%, which is 15.6% lower than that of BFRP. On the one hand, the film formed by the sizing agent on the surface of the basalt fiber will improve the adsorption of the fiber, strengthen its bond with the epoxy resin, prevent the transport of charge, and reduce conductivity loss. On the other hand, some flaws in the film will produce a mechanical anchoring effect after being filled with resin, improving the bonding strength of the interface and reducing the polarization loss by inhibiting the turning polarization of the resin long chain [25]. This also demonstrates that sizing agent B has the best impact on enhancing the insulating performance of basalt fiber/epoxy resin composites.

## 4. Conclusions

In this paper, three kinds of wetting agents with different composition and content were prepared, and the composite material was prepared with modified basalt fiber and epoxy resin. The surface morphology of the basalt fiber before and after modification was observed, and the thermal, mechanical, and insulation properties of the composite were explored. The following conclusions were reached:

(1) It can be observed by SEM that the sizing agent can form a film on the surface of the fiber, which can improve the sizing effect of the fiber and resin. However, when the concentration of the sizing agent is sufficiently high, the greatly increased bunching property will cause the phenomenon of binding between the fibers.
(2) The infiltration-modified composites’ mechanical characteristics showed various degrees of improvement. B-BFRP experienced increases in bending strength, bending modulus, tensile strength, and interlaminar shear strength of 122%, 34%, 102%, and 10.2%, respectively. Through DMA experiments, it was discovered that the addition of a sizing agent can lower the material’s elasticity and storage modulus while also reducing the Tg of the composite material.
(3) Compared with the unmodified BFRP, the insulation properties of the modified composites generally showed a trend of increasing first and then decreasing. Among them, sizing agent B has the best effect on improving the insulation performance of composite materials: the breakdown strength can be increased by 112%, and the leakage current and dielectric loss factor can be reduced by 25.4% and 15.6%, respectively.

## Figures and Tables

**Table 1:** Proportion of each component of sizing agent.

Number	Epoxy Emulsion Film- Forming Agent (wt%)	Acrylic Emulsion Film- Forming Agent (wt%)	Polyurethane Emulsion Film-Forming Agent (wt%)	Coupling Agent (wt%)	Lubricant (wt%)	Antistatic Agent (wt%)
A	5	2	0	1	0.4	0.1
B	5	2	1	1	0.4	0.1
C	5	2	2	1	0.4	0.1

## Data Availability

The original data needed to reproduce these discoveries cannot be shared, because these data are also part of the ongoing research. The original data used to support the results of this study can be obtained from the communication’s author.
